# Carbolic Acid as an Hypodermic Injection for the Cure of Hemorrhoids, Carbuncle, Etc.

**Published:** 1886-07

**Authors:** N. B. Kennedy

**Affiliations:** Hillsboro, Texas


### CARBOLIC ACID AS AN HYPODERMIC INJECTION FOR THE CURE OF HEMORRHOIDS, CARBUNCLES, ETC.

BY N. B. KENNEDY, M. D., HILLSBORO, TEXAS.

My attention was first directed to the hypodermic use of carbolic acid in the treatment of hemorrhoids as an hypodermic, in 1875, on account of its antiseptic anæsthetic, slightly caustic, and powerful disinfectant properties. It has a very slight claim to being an acid, but is more closely allied to alcohol, and in consequence is sometimes called *phenylic alcohol*. Carbolic acid has a most wonderful power of preventing putrefaction in animal substances, arresting fermentation and coagulating albumen; and its great curative power, in my opinion, is due to the fact of its coagulating the albumen of the blood. The U. S. Dispensatory says it is a powerful irritant when applied to the epidermis. I have in my limited experience found it only slightly so. When applied undiluted to the epidermis, it causes it to turn white, with but little pain, and in most cases no vessication whatever.

When I commenced the use, eleven years ago, of carbolic acid, hypodermically, it was with an abiding faith that I would succeed, and success has followed beyond my most sanguine expectations; and to-day, in the broad light of the gigantic advancement in science, I can safely affirm that the hypodermic administration of carbolic acid is the *sine qua non* of conservative surgery, and the knife will be dispensed with in many, if not in a majority of surgical operations.

During the eleven years I have used it, I have never witnessed any of its toxic effects—such as nausea, cold sweats, marked pallor of the skin, stupor, rapidly deepening into complete insensibility—a feeble pulse, which is generally rapid, and great disturbance of the breathing. I have never, from its use externally, met with the dark-colored urine mentioned by writers.

I have also used it quite extensively, as internal medicament (with the happiest effect), for its sedative effect upon the gastro-intestinal mucous membrane, and its anti-fermentative action upon the *primæ viæ*. It is an admirable remedy in vomiting and diarrhœa, caused by excessive irritability of the gastric or intestinal mucous membrane; in flatulence—yeasty vomiting—in diarrhœa with offensive discharges—in the fermentative diarrhœa connected with intestinal dyspepsia, one of our most valuable remedies when given in doses from one to three drops. It should not be given oftener than every three hours. The best menstruum is glycerine, which dissolves it in all proportions.

Since the commencement of its use in 1875, I have met with but one single failure, and that was a keloid tumor situated on the pectoral region. The tumor would very nearly disappear, and then return again as large as ever. It is true I had two hemorrhoidal tumors to slough, but they healed very kindly (with an entire disappearance of tumors) under the following treatment:

℞. Iodoform.

Oil Rose ............... *aa* ............... ℨ ss

Acid carbolic .......................... grs. xx

Glycerine (pure) .......................... ℨii

Ung. petrol. q. s. .......................... ℥i

M. ft. ungentum.

Apply to slough three or four times daily. The oil of rose almost completely masks the intense and disagreeable odor of the iodoform.

The following is a report of some few out of the many cases treated most successfully hypodermically and locally with carbolic acid.

*Case 1.*—March, 1875. Called to see Mrs. McA., a delicate little woman, whom I found suffering with an hemorrhoidal tumor nearly as large as a goose egg. She was suffering such intense agony that I determined to inject morphia and carbolic acid into the tumor. I injected five or six drops of Calvert's pure carbolic acid, made soluble in just as small a quantity of pure glycerine as would dissolve it, and a half grain of morphia, into the centre of the tumor as near as I could get it. The pain was very great, but subsided in less than two minutes, when there was an entire absence of all pain. Four more injections of the acid alone effected a complete cure in this case.

*Case 2.*—Called August 19, 1878, to see J. R. D., aet. 50, suffering with fifteen large carbuncles on his back and neck. He was suffering fearful agony, his family informing me he had not slept five minutes in five days and nights. I at once injected four or five drops of carbolic acid into each carbuncle with the happiest effects. In five minutes after injecting he was in a sound, refreshing sleep, which continued without intermission from 4 o'clock p. m. until 9 o'clock a. m. He awoke from sleep greatly refreshed and free from pain, and never did have any more pain. Twelve of the carbuncles aborted and three went on to suppuration, or rather had commenced to suppurate when I first saw them. A complete cure followed in three weeks. Before this he was frequently afflicted with carbuncles, but since he has never had a carbuncle, and to-day is in splendid, robust health and a happy man.

*Case 3.*—J. L. F., a well-to-do farmer, consulted me in reference to a tumor on the back of his neck which had commenced to grow and was paining him. I found a fibro-cellular tumor about the size of a small egg, and injected twenty drops of acid of full strength into the centre of the tumor, which in this case suppurated, but healed kindly in ten days and left no cicatrix behind.

*Case 4.*—December 10, 1880. Miss J. A., a young lady, aet. eighteen years, in the full bloom of womanhood and beauty; a very large tumor in the right mamma. Her history was good—had been in good health all her life. Two years since the *catamenia* was eslablished, synchronous with which she noticed a swelling in the right mamma. It had been growing for two years, and was now as large as a child's head. She had never suffered any pain in the gland. I diagnosed it to be a multilocular adenoid tumor. I injected about twenty drops of pure undiluted acid into the most prominent portion of the tumor. The pain was very slight, the anæsthetic effects of the acid following immediately the withdrawal of the needle. I used the remedy from week to week once a week for two or three months, at the expiration of which time the tumor had decreased in size from a child's head to a goose-egg. I now commenced its use twice a week, and soon reduced the tumor to the size of a hen's egg. At this time the young lady moved to a distant county. I learn the tumor entirely disappeared. She married—had a child—and found the right mamma unaffected and the secretion of milk as abundant as in the left.

*Case 5.*—A highly nervous young lady came to my residence to have a felon lanced. She was clamorous for chloroform, as she feared the pain would kill her. I bathed the finger in pure undiluted acid, and in two minutes lanced the felon almost without pain, much to her astonishment.

*Case 6.*—August 24, 1885. Mr. Isaac M. Newton, aet. about thirty-five, living at Mt. Calm, twenty-five miles distant, came to me to be treated. On an examination I found five hemorrhoidal tumors, the mass being as large as my two fists. The patient was cachetic and emaciated to an extreme degree. I injected about twenty drops into one tumor. In two hours he sent for me. I found the tumor strangulated and he was suffering excruciating agony. I placed him fully under chloroform and reduced it. As soon as he would get from under the influence of the anæsthetic, the tumor would protude again. I gave him one grain of morphia hypodermically, which quieted all pain. On the next day I injected twenty drops of the acid into another, and so on from day to day until I had injected all five, when the tumors had become so much reduced in size that they returned within the sphincter of their own accord. I continued the treatment for ten days, placing him on a good blood-making tonic, when he returned home, and inside of three weeks he was entirely restored to health and usefulness. He is a farmer, and can now do as much work as anyone.

*Case 7.*—Mr. C. A. J. W., a prominent merchant of our city, had three hemorrhoidal tumors as large as small hen eggs. I injected twenty drops of acid into one tumor, when he begged me to inject the other two, which I did—thus injecting sixty drops of the pure undiluted acid at one sitting, but there was not the slightest toxic effect; the tumors sloughed, however, but healed very kindly under treatment.

In conclusion, allow me to advance the opinion that in carbolic acid we have one of the most powerful remedies for good in the whole pharmacopœia.

